# Benefits of tunnel handling persist after repeated restraint, injection and anaesthesia

**DOI:** 10.1038/s41598-020-71476-y

**Published:** 2020-09-03

**Authors:** Lindsay J. Henderson, Bridgette Dani, Esme M. N. Serrano, Tom V. Smulders, Johnny V. Roughan

**Affiliations:** 1 Centre for Behaviour and Evolution, Newcastle University, Newcastle Upon Tyne, NE2 4HH UK; 2 Institute of Neuroscience, Newcastle University, Newcastle upon Tyne, NE2 4HH UK; 3 The Roslin Institute, The University of Edinburgh, Midlothian, EH25 9RG UK

**Keywords:** Animal behaviour, Behavioural methods

## Abstract

Millions of mice are used every year for scientific research, representing the majority of scientific procedures conducted on animals. The standard method used to pick up laboratory mice for general husbandry and experimental procedures is known as tail handling and involves the capture, elevation and restraint of mice via their tails. There is growing evidence that, compared to non-aversive handling methods (i.e. tunnel and cup), tail handling increases behavioural signs of anxiety and induces anhedonia. Hence tail handling has a negative impact on mouse welfare. Here, we investigated whether repeated scruff restraint, intraperitoneal (IP) injections and anaesthesia negated the reduction in anxiety-related behaviour in tunnel compared with tail handled BALB/c mice. We found that mice which experienced repeated restraint spent less time interacting with a handler compared to mice that were handled only. However, after repeated restraint, tunnel handled mice showed increased willingness to interact with a handler, and reduced anxiety in standard behavioural tests compared with tail handled mice. The type of procedure experienced (IP injection or anaesthesia), and the duration after which behaviour was measured after a procedure affected the willingness of mice to interact with a handler. Despite this, compared with tail handling, tunnel handling reduced anxiety in standard behavioural tests and increased willingness to interact with a handler within hours after procedures. This suggests that the welfare benefits of tunnel handling are widely applicable and not diminished by the use of other putatively more invasive procedures that are frequently used in the laboratory. Therefore, the simple refinement of replacing tail with tunnel handling for routine husbandry and procedures will deliver a substantial improvement for mouse welfare and has the potential for improving scientific outcomes.

## Introduction

The laboratory environment and routine handling can increase anxiety in research animals, impeding their health and welfare^1–4^. The standard method used to capture and handle laboratory mice is to pick up and grasp the mouse by its tail^5–8^. Tail handling only requires mice to be suspended by their tail for a few seconds, before being supported on the hand or arm of the handler. Despite this, tail handling can negatively influence the behaviour of mice and reduce their willingness to interact with handlers^9–11^. In recent years, alternative non-aversive methods for picking up mice have been investigated and validated^9–11^. Namely, tunnel handling, which involves guiding mice into a tunnel before being lifted, and cup handling, where mice are scooped up and lifted with closed or open hands^9,11^. If refinement of handling methods can reduce background stress and anxiety in laboratory animals, this could significantly improve the welfare of millions of mice that are used every year for scientific research. Furthermore, this refinement has the potential to reduce data variability caused by handling stress^12^, reducing the number of animals required for experiments and improving the reproducibility and replicability of results.

There is mounting evidence that picking mice up in a tunnel or cupped hands, rather than by the tail reduces anxiety, increases willingness to interact with a handler and enhances the performance of mice in standard behavioural tests^9–14^. These findings have been replicated in several laboratories and have confirmed that the influence of handling methods upon behaviour are highly consistent under both the light and dark cycle, across handlers and with different strains of mice^9–14^. Furthermore, handling method can impact physiological indices^13,15^ and tail handled mice show decreased responsiveness to reward compared to tunnel handled mice, indicative of anhedonia and chronic stress^14^. Importantly, single or repeated restraint^9,12,16^, a single intraperitoneal (IP) injection or repeated subcutaneous injection^12,16^, repeated oral gavage^12^, and tattooing or ear-tagging^17^ for identification, do not negate the beneficial effects of tunnel handling upon voluntary interaction with a handler. Mice handled using a tunnel also show reduced facial grimace scores compared to tail handled mice, suggesting they may be less susceptible to pain^17^. This suggests the experience of putatively more invasive procedures that are frequently used in the laboratory appear not to negate the benefits of tunnel handling. While the behavioural impacts of non-aversive handling have not been replicated in every aspect in previous studies (review of current research here; https://www.nc3rs.org.uk/mouse-handling-research-papers), the majority of studies provide substantial evidence that using non-aversive handling methods in place of picking up mice by the tail can increase ease of handling for researchers and animal care staff, reduce the anxiety caused by handling, and improve the welfare of laboratory mice^12,16^.

Experimental protocols vary across studies and laboratories, and often require mice to undergo repeated procedures that can cause pain or discomfort. The frequency of injections can vary; for example, daily injections over a short duration, or weekly injections over a longer period. Mice are also commonly held under anaesthesia in the laboratory for short durations, for the implantation of devices^18^, for serial imaging or for injection of noxious agents^19^. Alternatively, mice are held under anaesthesia for a longer duration for more extensive surgical procedures^20^. Validating the efficacy of handling methods at reducing handling stress and anxiety across these varied experimental protocols is relevant to real-world scenarios within the biomedical fields, and may facilitate the uptake of non-aversive handling methods across laboratories^8^. Moreover, whether anaesthesia or repeated IP injection influences the impact of handling methods upon mouse behaviour and anxiety is yet to be addressed.

This study aims to further elucidate whether the benefits of tunnel handling persist after repeated restraint, intraperitoneal (IP) injection or short duration anaesthesia. Firstly, we assessed the influence of repeated scruff restraint (daily for 4 days) compared with controls that were only picked up by tunnel or tail handling methods, upon voluntary interaction with a handler and behaviour in standard tests, the open field test and the elevated plus maze (Experiment 1). We compared two scruff restraint methods; the standard method of pinching the loose skin of the neck between the thumb and forefinger to immobilise the animal in the hand, and a potentially refined method, where the loose skin of the neck is grasped between the thumb and middle finger, and the forefinger provides support at the back of the head, reducing tension across the throat of the mouse (Illustrated here: https://norecopa.no/scruff). We then tested the behavioural response of mice that had been tail or tunnel handled to repeated restraint and IP injection (Experiment 2), or repeated short duration anaesthesia with isoflurane (Experiment 3). Based on the existing evidence^9,12,16^, we predicted that the benefits of tunnel handling upon willingness to interact with a handler and reduced behavioural anxiety in standard tests would persist after repeated restraint, IP injections and anaesthesia.

## Methods and materials

### Animals, housing and husbandry

Forty-eight mice of both sexes (BALB/c) were purchased from Charles River Laboratories, UK and were between 4 and 7 weeks of age on arrival. For the restraint experiment (Experiment 1), mice were run in two sequential batches (N = 24 per batch, October and December 2018). To minimise animal use, the mice from the October batch (N = 20) went on to be used in Experiment 2 (IP injection) and those from the December batch (N = 24) were used in Experiment 3 (anaesthesia). After completion of Experiment 1 and before the beginning of the IP injection experiment, two cages of male mice (one tunnel handled and one tail handled), showed signs of escalated aggression and were humanely euthanized by cervical dislocation. Experiments 2 and 3 were run concurrently, and mice were run in two batches (N = 10 or 12 per experiment per batch, January–February then February–March 2019). For the duration, mice were pair-housed in IVC 420 cages (160 mm (w) × 339 mm (l) × 130 mm (h), Arrowmight), with sawdust bedding and nesting material (4HK Aspen chips, NestPak and Sizzlepet nesting, Datesand Ltd, Manchester). All cages had a clear Perspex home-cage tunnel (50 mm diameter, 150 mm length). Animals had access to food (Special Diet Services, RM3E diet) and water ad libitum, and were cleaned approximately once per week. The holding rooms were kept at a relatively constant temperature (range 21–23 °C) and humidity (range 45–50%). Mice were maintained on a 12:12 h light/dark cycle (lights on at 07:00) and experiments were conducted between 10:00 and 16:00. Handling method remained the same for all mice across the experiments, and handling methods and sex were balanced across batches and experiments. During experiments mice were held in a single room. Between experiments mice were moved to a larger holding room (under the same photoperiod) that houses the mouse colony at Newcastle University, UK. During this time mice were only handled using their assigned handling method (tail or tunnel) during weekly cage cleaning. Prior to the beginning of Experiment 1, a week after habituation to the laboratory, mice were marked for identification using hair dye (Just for Men, moustache and beard, Real black M55, UK). Hair dye has previously been shown to not interfere with the response to handling^9,11,14^; mice did not need to be re-dyed prior to Experiments 2 and 3, as marks were still visible. For Experiments 2 and 3, mice were moved back from the colony housing (where they had been housed for 4 weeks (Exp. 3) or 8 weeks (Exp. 2)) and habituated to the room in which experiments were conducted, for one week.

### Handling methods

Each cage of two mice was randomly assigned to one of two handling methods, tail or tunnel handled (sexes were equally split between the handling methods). The animals were not handled regularly during habituation before experiments, but if handling was necessary their respective handling method was used. Mice were handled by their designated method by a handler wearing nitrile gloves. The handler first handled the soiled bedding in the cage before each handling session, as the nest material was removed from the cage. Gloves were changed between each cage. Cages were counterbalanced across the day, with respect to handling method and sex. Tail handling involved grasping a mouse at the base of its tail using the thumb and forefinger, and then lifting onto the sleeve of the laboratory coat for 30 s (suspended by tail for less than 2 s) before being returned to its home cage. For tunnel handling, the mouse was guided into the Perspex tunnel, and lifted above the cage and held for 30 s. The handler’s hands were loosely cupped over the ends of the tunnel to prevent escape. For daily handling during experiments, mice were handled twice daily for 30 s, 60 s apart. During the handling sessions, the occurrence of any urination or defecation when a mouse was picked up and held was recorded. For routine husbandry practices, such as cage cleaning, mice were captured and transferred using their designated handling method either on the sleeve for tail handled mice, or in the tunnel for tunnel handled mice. The same protocol was used when transferring mice to and from behavioural tests, i.e. the elevated plus maze and open field test. Three female handlers conducted all handling and behavioural tests (LJH Exp. 1, EMNS Exp. 2 and BD Exp. 3), JVR handled mice for anaesthesia (Exp. 3).

### Behavioural tests

The methods used to assess behavioural anxiety were the elevated plus maze (EPM), and open field test (OFT). In addition, we used voluntary interaction (VI) tests, which provide a measure of the willingness of mice to approach their handlers. The VI test has been shown to be a robust measure of anxiety in anticipation of, and post handling^9,11,16^. Unlike the EPM and OFT, it is less susceptible to habituation issues^10^. Experiments were designed to ensure mice received no more than one repetition of either the OFT or EPM but had multiple VI tests. The procedures used for the VI tests, EPM and OFT were informed by previous studies^9,14,16^.

### Voluntary interaction tests

For all tests, the nesting material and the home cage tunnel were removed from the cage, and the handler stood motionless in front of the cage for 60 s. Gloved hands were then held resting on the substrate in the front of the cage for 60 s to assess voluntary interaction. Behaviour was filmed from 200 mm above using a video camera mounted on an extension arm fitted to a tripod, and the recordings were later analysed. Time spent interacting with the handler was measured for each mouse within a cage, from which an overall mean cage score was calculated. Therefore, for VI tests, the experimental unit was ‘cage’. Interaction was defined as sniffing (nose within 5 mm), touching (including paw contact), and climbing on the hand. For Experiment 1, VI tests were done both immediately before and after handling. Whereas, for Experiments 2 and 3, mice only underwent VI tests after handling, not before. Three observers that were blind to the handling method of the mice completed the video analysis. The data from video analysis were highly repeatable between observers^21^ (n = 4, *r* > 0.80,* P* < 0.001).

### Elevated plus maze and open field test

The EPM was elevated 350 mm from the ground and had arms measuring 50 mm (w) × 300 mm (l) with side walls of 150 mm on the two closed arms. Using their assigned handling method mice were placed into the centre of the maze facing an open arm and filmed for 5 min. For the OFT each mouse was individually placed via their designated handling method into the centre of a rectangular arena made of grey plastic (600 mm (w) × 700 mm (l) × 550 mm (h)) and filmed for 5 mins. The first mouse to undergo testing from its cage was returned to an empty holding cage briefly, to avoid any impact upon the behaviour of the cagemate. Whereas, the last mouse tested from each cage was returned to its home cage immediately after testing. The OFT and EPM were cleaned with 70% ethanol after each observation, and the running order of mice was counterbalanced across the testing day, with respect to handling method, treatment and sex.

For the EPM and OFT behaviour was filmed and later analysed. For the EPM the number of entries into the open arms of the maze and time spent in the open and closed arms of the maze was scored. Entry into, and time spent in an arm was defined as being when all four paws were in the arm. For the OFT, time spent in the centre of the arena, number of entries into the centre of the OFT was scored. The centre of the arena was defined as being > 100 mm from the edge. The number of faecal boli produced by mice in both the EPM and OFT was recorded. A single treatment blind observer completed the OFT video analysis. Three observers blind to the handling method of the mice completed the video analysis for the EPM. The data from video analysis were highly repeatable between observers^21^ (n = 4, *r* > 0.96,* P* < 0.001).

### Experiment 1: restraint

**Figure 1:**
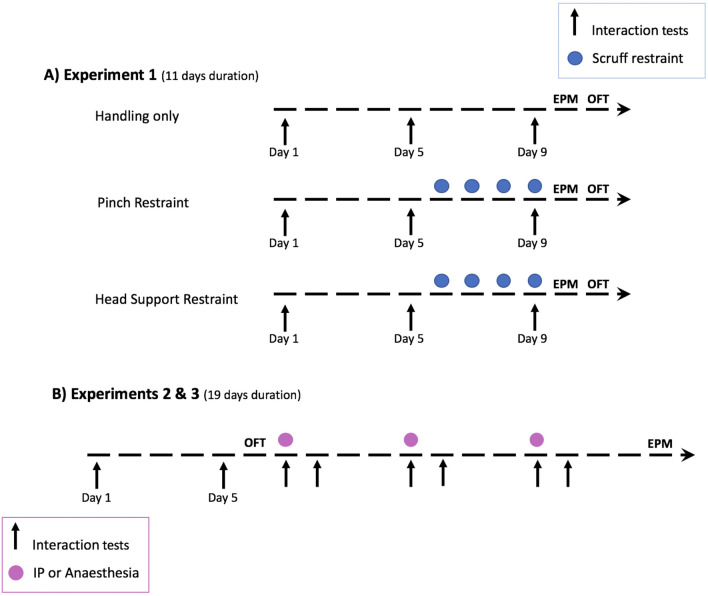
Study designs and timelines for experiments (BALB/c mice, N = 48 mice). (**A**) Experiment 1: Mice were equally split between tail or tunnel handling methods, and then divided into three experimental groups (N = 8 mice per handling method and restraint group). Handling only; mice experienced their designated handling method daily for 9 days. Pinch Restraint; handling only day 1–5, then restraint on days 6–9. Head Support Restraint; handling only day 1–5, then restraint on days 6–9. Voluntary interaction tests were conducted on day 1, 5 and 9. All mice were then tested in an elevated plus maze (EPM) and an open field test (OFT). (**B**) Experiments 2 and 3: Tail and tunnel handled mice were split into two experiments; IP injection (N = 20 mice, N = 10 mice per handling method) or anaesthesia (N = 24 mice, N = 12 mice per handling method). For Experiments 2 and 3 all mice experienced handling only for the first 5 days followed by an OFT on day 6. Mice were then anaesthetised or received an IP injection on three occasions, three days apart (day 7, 11 and 15). Voluntary interaction tests were conducted on day 1 and 5, and immediately after each procedure, and on the following day immediately after handling. This was followed by a final EPM test.

This experiment was designed to investigate whether repeated restraint negated the reduction in anxiety-related behaviour in tunnel compared with tail handled mice. As shown in Fig. 1A, mice initially underwent a period of daily tail or tunnel handling, and VI tests were conducted on day 1 and day 5. To assess the response to repeated restraint the mice were split into three treatment groups within each handling method. (i) Handling only; mice were picked up daily using their designated handling method (tail or tunnel) from day 1–9 (Fig. 1A). For the other two treatment groups mice were picked up daily using their designated handling method (tail or tunnel) from day 1–5, then restrained daily for four days from day 6–9 (Fig. 1A). Two methods commonly used to restrain mice were tested. After mice were captured using their familiar method (tail or tunnel) and placed on the bars of a clean cage top, mice were restrained by; (ii) holding the tail in place, and pinching the loose skin of the neck between the thumb and forefinger to immobilise the animal in the hand (Pinch Restraint). Or (iii) restrained by holding the tail in place and grasping the loose skin of the neck between the thumb and middle finger, the forefinger was then placed on the back of the head to immobilise the animal in the hand (Head Support Restraint). For both restraint methods, the mouse was then held on its back above the home cage for 10 s before being released back into the cage. To assess the influence of restraint upon voluntary interaction with a handler, all mice also underwent VI testing on day 9 (Fig. 1A). On day 10, mice were recorded in the EPM, followed by the OFT on day 11. Mice were not handled on these days, apart from when they were transferred to and from the test arenas. The experimental treatments were counterbalanced across the sexes, within each cage both mice experienced the same treatment.

### Experiment 2: IP injection

This experiment was designed to assess whether experience of repeated IP injection affected the behaviour of mice that experienced different handling methods. As mice had not undergone any formal handling for several weeks (only weekly cage cleaning), the experiment began with five days of daily handling. To establish whether handling method (tail vs. tunnel) continued to influence voluntary interaction with the handler, mice had VI tests on day 1 and day 5, prior to the start of procedures (Fig. 1B). In addition, mice underwent an OFT prior to the start of procedures on day 6 (Fig. 1B). Starting from day 7, all mice received an IP injection on three occasions, three days apart (day 7, 11 and 15, Fig. 1B). Each mouse was given an IP injection of saline at 14 ml/kg (70% of the maximum volume recommended by LASA^22^) using a 29-gauge needle. Briefly, each mouse was lifted by its designated handling method onto a clean cage top from its home cage, mice were then restrained by grasping the tail in one hand, and the loose skin of the neck between the thumb and forefinger in the other hand to immobilise the animal (Pinch Restraint). The mouse was then held on its back for injection. The injection site was alternated between left and right side across injections for each mouse. Care was taken not to lift the mouse by the tail during restraint for tunnel handled mice. Any urination or defecation produced during the injection procedure was counted. To determine the influence of IP injection upon willingness to interact with handler, mice underwent VI testing immediately after each injection, and then a day later immediately after handling (Fig. 1B). Finally, mice were recorded in an EPM after they had experienced the procedures (Fig. 1B). The running order was counterbalanced with respect to handling method and sex, across the procedure and testing days.

### Experiment 3: anaesthesia

This experiment was designed to assess whether experience of repeated anaesthesia affected the behaviour of mice that experienced different handling methods. As mice had not undergone any formal handling for several weeks (only weekly cage cleaning), the experiment began with 5 days of daily handling (as in Experiment 2). To establish whether handling method (tail vs. tunnel) continued to influence voluntary interaction with the handler, mice had VI tests on day 1 and day 5, prior to the start of procedures (Fig. 1B). In addition, mice underwent an OFT prior to the start of procedures on day 6 (Fig. 1B). Starting on day 7, each cage of mice was anaesthetised on three occasions, three days apart (day 7, 11 and 15, Fig. 1B). Home cage pairs of mice were placed into an induction chamber using their designated handling method. Mice first received 1 min of oxygen, followed by 4% isoflurane at a flow rate of 2–3 l. Approximately 5 s after the last purposeful movement made by either mouse, both were considered to be sufficiently anaesthetised and were moved to a heat pad (36–38ºC) and maintained under anaesthesia for a further 5 min via face mask delivery of 2–3% isoflurane in 2L/min oxygen. Mice were then moved to an empty cage lined with paper towels for recovery (160 mm (w) × 339 mm (l) × 130 mm (h)), for 15 min where they were monitored. Following this, mice were returned to their home cage for 10–20 min (mice were fully mobile at this time). Mice then underwent post-procedural VI testing, which was repeated 24 h later immediately after handling (Fig. 1B). Any urination or defecation produced in the induction chamber, on the heat pad or in the recovery chamber was recorded. Finally, mice were recorded in an EPM after they had experienced the procedures (Fig. 1B). The running order was counterbalanced with respect to handling method and sex, across the procedure and testing days.

### Statistical analysis

All analyses were conducted in R 3.3.2 (R Development Core Team, 2016^23^), using the car, MASS and lme4 packages. Datasets were tested for normality and homogeneity of variance, where assumptions were not met, data were transformed or appropriate error structures were used^24^. Where significant main effects were found, post-hoc tests were performed to examine pairwise comparisons. For full details of statistical tests see Supplementary Materials 1, and for results of full statistical models see tables in Supplementary Materials 2.

### Experiment 1

To investigate whether handling method, acute handling experience and number of handling days influenced voluntary interaction with the handler we used General Linear Mixed Models (GLMMs). Proportion of time spent interacting with the handler was the dependent variable, and handling method (tail vs. tunnel), order (pre or post handling), and day of observation (1, 5 or 9) were the independent variables. All interactions were fitted. To examine the effects of our restraint treatments upon voluntary interaction with the handler on day 9, a General Linear Model (GLM) was used. Proportion of time spent interacting with the handler was the dependent variable, and handling method (tail vs. tunnel), restraint treatment (Pinch Restraint, Head Support Restraint or handling only) and their interaction, were the explanatory variables. In these models, only observations taken after handling were included. GLMMs with a Poisson error structure, were used to analyse whether handling method and restraint treatment influenced entries into the open arm of the EPM, and entries into the centre of the OFT. This is because these data were a count. A GLMM was also used to examine whether handling method and restraint treatment affected time spent on the open arm of the EPM and time spent in the centre of the OFT. A square root transformation was performed to normalize proportion of time spent interacting with the handler, and proportion of time spent in the centre of the OFT. An Arcsine transformation was performed to normalize proportion of time spent on the open arm of the EPM. To examine whether handling method influenced incidence of defecation throughout handling and the behavioural tests, a GLMM with a Poisson error structure was used. A GLM was used to analyse whether mass measured at the beginning and end of the experiment was influenced by handling methods. For models with repeated measures or mouse as the experiment unit, cage was included as a random effect to avoid pseudoreplication.

### Experiments 2 and 3

We used GLMMs to investigate whether handling method (tail vs. tunnel) and handling day (day 1 or day 5) influenced voluntary interaction with the handler’s hand prior to starting procedures. Proportion of time spent interacting with the handler was the dependent variable, and handling method (tail vs. tunnel) and day of observation (1 or 5) were the explanatory variables. All interactions were fitted. GLMMs were used to examine whether procedures affected voluntary interaction with the handler on the day of the procedure or the day after. The dependent variable was the proportion of time spent interacting with the handler, which was calculated as a mean per cage over the three tests. Handling method (tail vs. tunnel), Day (day of procedure or day after procedure), procedure (anaesthesia or IP) and their interactions, were the explanatory variables. The GLMMs used to analyse the OFT and EPM data were the same as outlined above for Experiment 1. However, the explanatory variables were handling method (tail vs. tunnel) and procedure (anaesthesia or IP). To examine whether handling method influenced incidence of defecation throughout handling and the behavioural tests, a GLMM with a Poisson error structure was used. A GLM was used to analyse whether mass measured at the beginning and end of the experiment was influenced by handling methods. For all models, cage was included as a random effect to account for repeated measures and to avoid pseudoreplication.

### Ethical statement

Experiments were conducted at Newcastle University following approval from the University’s Animal Welfare and Ethical Review Body. Animal use and husbandry was in accordance with EU directive 2010/63/EU and UK Home Office code of practice for the housing and care of animals bred, supplied and used for scientific purposes. Procedures were conducted under UK Home Office licence, under the Animals in Scientific Procedures Act 1986 (PPL: P53E535ED). All animals were checked daily, and no adverse effects were reported during experiments. At the end the experiments the animals underwent terminal isoflurane anaesthesia followed by cardiac puncture and exsanguination. Death was then confirmed by cervical dislocation in accordance with Schedule 1.

## Results

### Experiment 1: restraint

#### Voluntary interaction with handler

**Figure 2:**
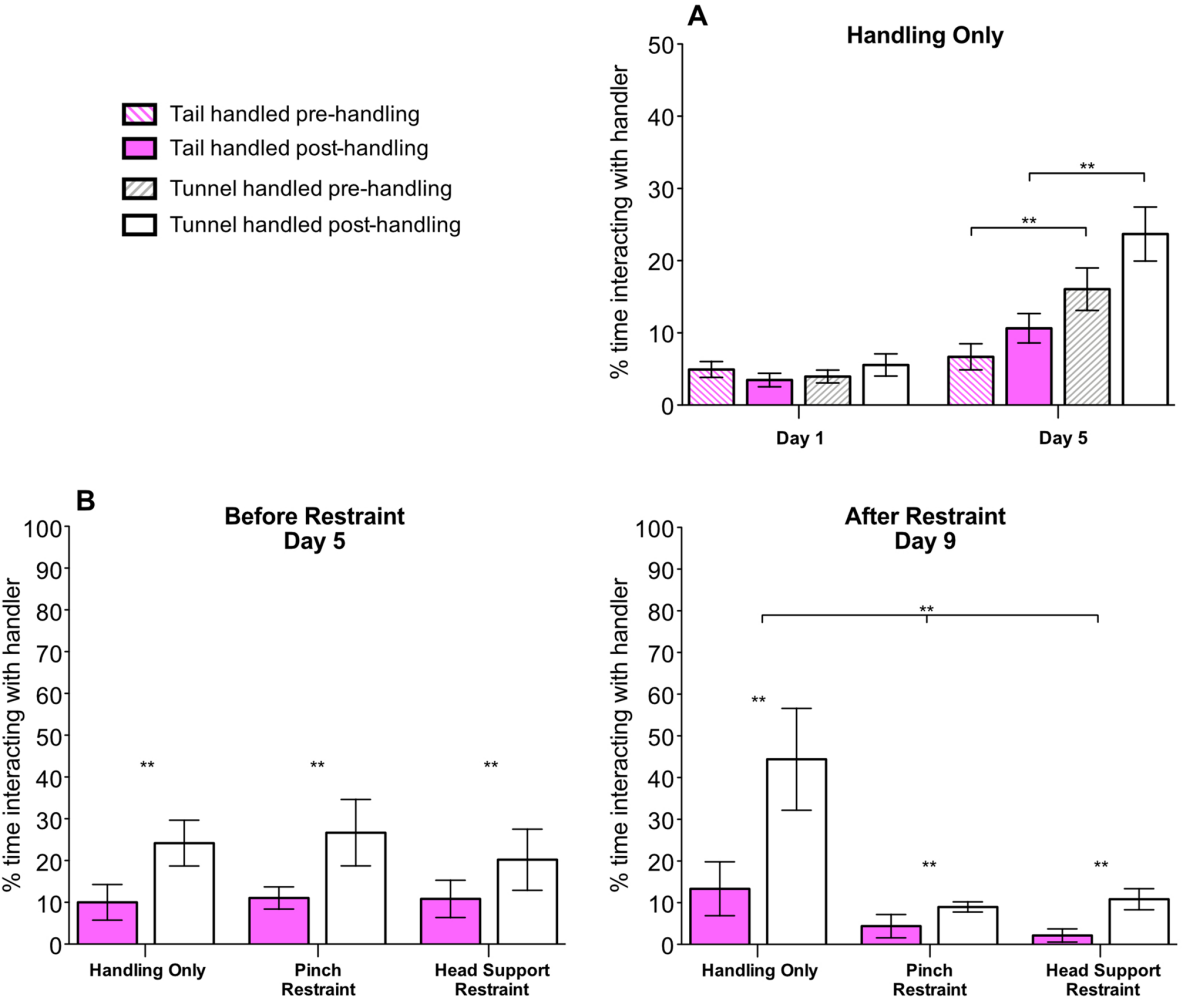
Effect of handling method and restraint upon time spent interacting with the handler (N = 24 cages, mean ± 1SEM). (**A**) Voluntary interaction (VI) tests were conducted on two days; day 1 the first day of daily handling and day 5 after five days of handling. VI tests were conducted both before (pre-) and after (post-) the animals were handled via either tail or tunnel handling. (**B**) Time spent interacting with the handling between treatment groups immediately after restraint or handling only, on day 5 before daily restraint began and on day 9. Mice were either handled only using tail or tunnel methods (handling only) on day 1–9 or were handling from day 1–5, then restrained daily using two methods of restraint (Pinch or Head Support) from day 6–9. ** denotes significant effects where *P* < 0.01.

Time spent interacting with the handler was influenced by both the handling method and the day of observation (Handling method × Day; ***χ***^2^ = 11.76, *P* < 0.01; Fig. 2A,B, Table 1A). Tunnel handled mice spent more time interacting with the handler than tail handled mice on day 5 (***χ***^2^ = 10.17, *P* < 0.001) and day 9 (***χ***^2^ = 9.46, *P* = 0.002), but not day 1 (***χ***^2^ = 0.02, *P* = 0.88). There was a trend that mice interacted more with the handler after handling compared with before (***χ***^2^ = 3.77, *P* = 0.05). In addition, there was a non-significant trend that tunnel handled mice spent more time interacting with the handler after handling, compared with tail handled mice (Handling method × Order; ***χ***^2^ = 2.81, *P* = 0.09; Fig. 2A).

**Table 1:** (A) Results of GLMM investigating the influence of Handling Method (tail vs. tunnel), Order (pre or post handling), Day (observation day 1,5, or 9) and their interactions upon time interacting with the handler. (B) GLM investigating the effect of Handling Method and Restraint Treatment (handling only, Pinch Restraint, Head Support Restraint) upon voluntary interaction with the handler on day 9. N = 24 cages.

Factor	*χ*^2^	*d.f*	*P*
**(A) Time spent interacting with the handler**			
Handling	12.01	1	** < 0.001**
Order	3.77	1	0.05
Day	42.38	2	** < 0.001**
Handling × day	11.76	2	** < 0.01**
Order × day	2.66	2	0.26
Handling × order	2.81	1	0.09
**(B) Time spent interacting with the handler (Day 9)**			
Handling	16.49	1	** < 0.001**
Treatment	21.52	2	** < 0.001**
Handling × treatment	1.30	2	0.52

*P* values < 0.01 are highlighted in bold.

On day 9, tunnel handled mice spent significantly more time interacting with the handler than tail handled mice, whether they had experienced repeated restraint or handling only (Handling method: ***χ***^2^ = 16.49, *P* < 0.001; Restraint × Handling method: ***χ***^2^ = 1.30, *P* = 0.52; Fig. 2B, Table 1B). However, mice that experienced either method of restraint spent less time overall interacting with the handler compared with mice that had experienced handling only (***χ***^2^ = 21.52, *P* < 0.001, Fig. 2B). The influence of treatment group upon behaviour was not evident on day 5, before daily restraint began (***χ***^2^ = 0.38, *P* = 0.80, Fig. 2B).

### Elevated plus maze and open field test

**Figure 3:**
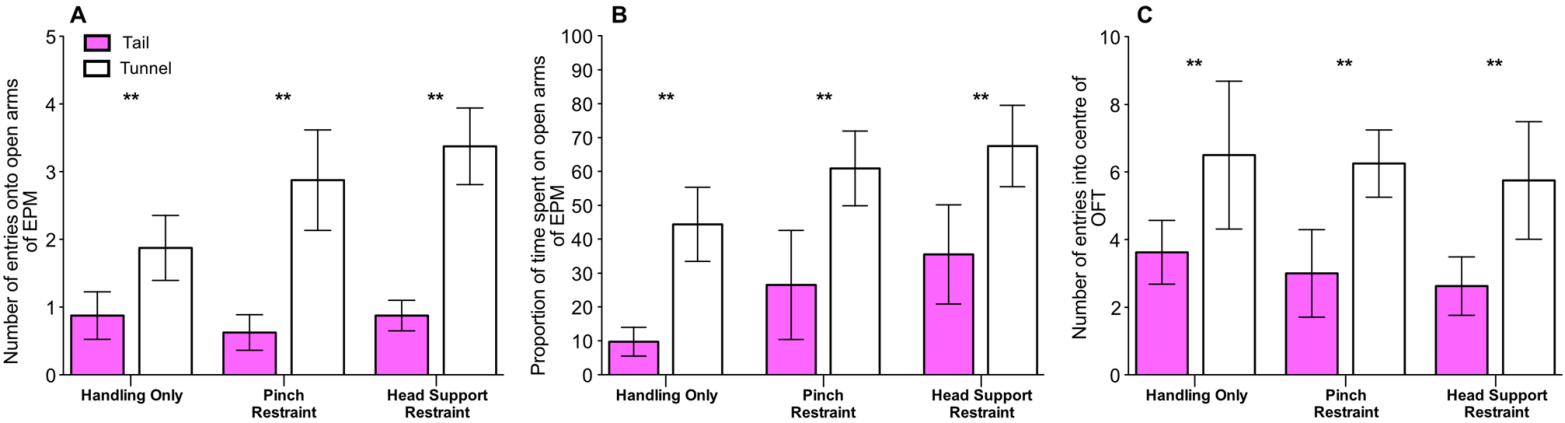
Influence of handling methods upon behaviour in the elevated plus maze (EPM) and open field test (OFT) (N = 48 mice, mean ± 1SEM). (**A**) number of entries onto the open arms of the EPM, (**B**) proportion of time spent in the open arms of the EPM, and (**C**) number of entries into the centre of the OFT, by tail and tunnel handled mice, that experienced three treatments; Handling only, Pinch Restraint or Head Support Restraint. ** denotes significant effects where *P* < 0.01.

Consistent with the prediction that tail handling produces higher levels of anxiety than tunnel handling, tail handled mice showed fewer entries onto the open arms of the EPM (***χ***^2^ = 20.99, *P* < 0.001; Fig. 3A), and spent less time on the open arm of the EPM than tunnel handled mice (***χ***^2^ = 4.28, *P* = 0.04; Fig. 3B). This result was unaffected by experience of restraint (Restraint treatment × Handling method: Entries, ***χ***^2^ = 1.48, *P* = 0.48, Time in open arm, ***χ***^2^ = 0.01, *P* = 0.99; Fig. 3A,B). For the OFT, tunnel handled mice entered the centre of the arena significantly more often than tail handled mice (***χ***^2^ = 12.62, *P* < 0.001; Fig. 3C). However, there was no difference in the amount of time spent in the centre of the OFT between the handling methods (***χ***^2^ = 1.99, *P* = 0.16). Experience of restraint did not influence the number of entries into the centre of the arena (Restraint treatment × Handling method: ***χ***^2^ = 0.25, *P* = 0.88; Fig. 3C), or time spent in the centre of the OFT arena (Restraint treatment × Handling method: ***χ***^2^ = 0.96, *P* = 0.62).

### Mass and defecation

There was no difference in mass between mice that experienced tail or tunnel handling at the beginning (Mean ± 1SEM: Tail = 20.6 ± 0.5 g, Tunnel = 21.1 ± 0.5 g, Day 0: *t* = 1.08, *P* = 0.29) or end of Experiment 1 (Mean ± 1SEM: Tail = 20.9 ± 0.5 g, Tunnel = 21.6 ± 0.5 g, Day 10: *t* = 1.30, *P* = 0.20). Combining all handling and behavioural tests, mice that were tail handled defecated more often than mice that were tunnel handled (***χ***^2^ = 8.70, *P* = 0.003).

### Experiments 2 and 3: IP injection and anaesthesia

#### Handling only phase

**Figure 4:**
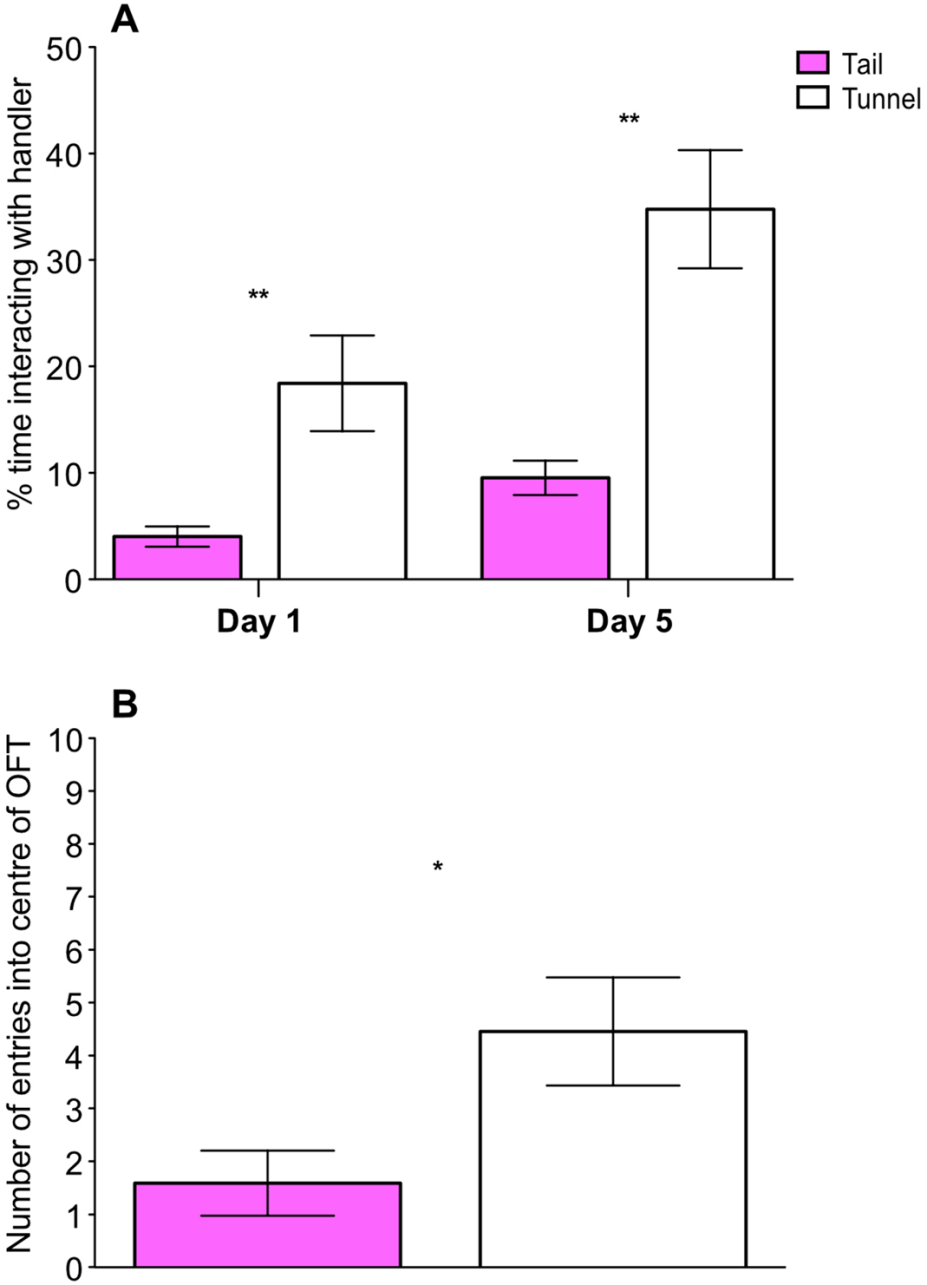
Influence of handling method upon voluntary interaction with a handler and behaviour in the open field test (OFT). (**A**) Time spent voluntarily interacting with the handler after a 4–8 week break between experiments on two days; day 1, the first day of daily handling and day 5, after five days of handling. Voluntary Interaction tests were conducted after the animals were handled via either tail or tunnel method (N = 22 cages). (**B**) Number of entries into the centre of the OFT by tail and tunnel handled mice (N = 44 mice). Graphs show mean ± 1SEM. * denotes significant effects where *P* < 0.05, ** denotes *P* < 0.01.

To establish whether the effect of handling method upon voluntary interaction with a handler was still evident after the gap between the experiments, mice were tested again after one and five days of daily handling. Time spent interacting with the handler was influenced by the handling method and day of observation. Tunnel handled mice spent more time interacting with the handler than tail handled mice on both day 1 and 5 (Handling method: ***χ***^2^ = 17.14, *P* < 0.001; Handling method × Day: ***χ***^2^ = 1.12, *P* = 0.29; Fig. 4A). Also, both tunnel and tail handled mice spent more time interacting with the handler on day 5 compared to day 1 (Day: ***χ***^2^ = 27.67, *P* < 0.001; Fig. 4A). Previous experience of restraint in Experiment 1 did not influence voluntary interaction with a handler (***χ***^2^ = 0.38, *P* = 0.83).

At the end of the handling only phase, tail handled mice continued to show fewer entries into the centre of the OFT compared with tunnel handled mice (***χ***^2^ = 5.49, *P* = 0.02; Fig. 4B). However, handling method did not influence the amount of time spent in the centre of the OFT (***χ***^2^ = 3.26, *P* = 0.07).

### Post-procedures

**Figure 5:**
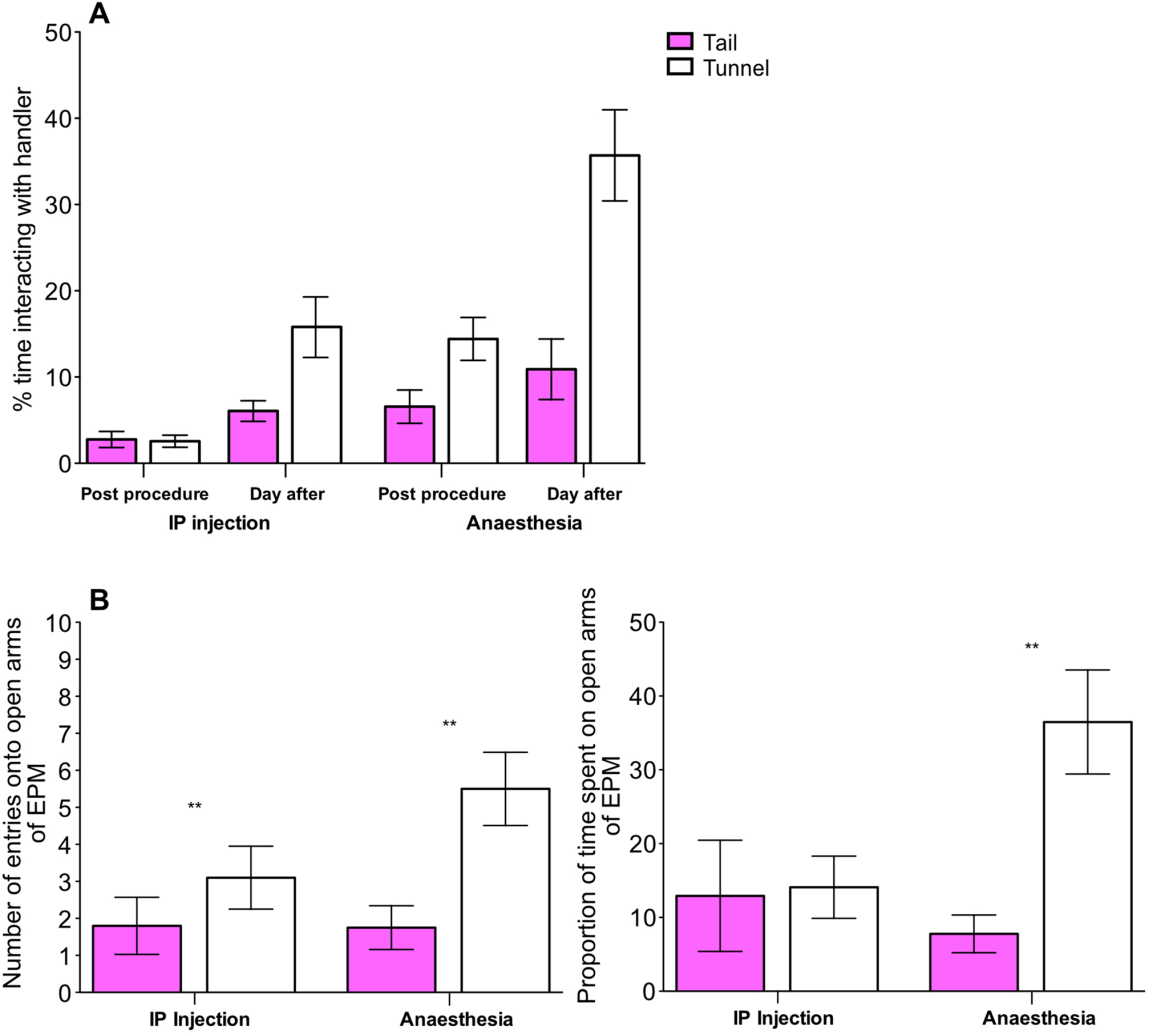
Effect of handling method and procedures (IP injection or anaesthesia) upon voluntary interaction with a handler and behaviour on the elevated plus maze (EPM). (**A**) time spent voluntarily interacting with the handler over three tests conducted immediately post-procedure and the day after IP injections or anaesthesia (N = 22 cages). (**B**) Number of entries onto the open arm and proportion of time spent on the open and closed arms of the EPM by tail and tunnel handled mice, that experienced either IP injections or anaesthesia (N = 44 mice). Graphs show mean ± 1SEM. ** denotes significant effects where *P* < 0.01.

Time spent interacting with the handler was influenced by the handling method, procedure type (IP injection or anaesthesia) and whether voluntary interaction was measured immediately after the procedure or the following day. Overall, tunnel handled mice interacted more with a handler than tail handled mice (***χ***^2^ = 16.15, *P* < 0.001). However, tunnel handled mice that underwent IP injection or anaesthesia, spent significantly more time interacting with the handler than tail handled mice on the day following the procedure, compared with immediately after the procedure (Handling method × Day, ***χ***^2^ = 8.92, *P* < 0.01; Fig. 5A). Also, tunnel handled mice that underwent anaesthesia spent significantly more time interacting with the handler than tunnel handled mice that underwent IP injection (Handling method × Procedure, ***χ***^2^ = 3.97, *P* = 0.05; Fig. 5A). The three-way interaction was not significant (*P* = 0.85).

Tail handled mice showed fewer entries onto the open arms of the EPM (***χ***^2^ = 8.12, *P* = 0.004; Fig. 5B), and this result was unaffected by whether mice experienced IP injection or anaesthesia (Handling method × Procedure, ***χ***^2^ = 0.56, *P* = 0.45; Fig. 5B). However, there was a trend that procedure type influenced whether handling method affected the proportion of time spent on the open arm of the EPM (Handling method × Procedure; ***χ***^2^ = 3.80, *P* = 0.05; Fig. 5B). Post-hoc tests showed that tail handled mice that underwent anaesthesia spent less time on the open arm of the EPM than tunnel handled mice (***χ***^2^ = 19.18, *P* < 0.001; Fig. 5B). Whereas, for mice that underwent IP injections, handling method did not influence the time spent on the open arm of the EPM (***χ***^2^ = 0.28, *P* = 0.59; Fig. 5B).

### Mass and defecation

There was no difference in mass between mice that experienced tail or tunnel handling at the beginning (Mean ± 1SEM: Tail = 25.5 ± 0.7 g, Tunnel = 26.8 ± 0.8 g, Day 0: ***χ***^2^ = 0.97, *P* = 0.32). There was a trend that tunnel handled mice were heavier than tail handled mice at the end of experiments 2 and 3 (Mean ± 1SEM: Tail = 26.0 ± 0.7 g, Tunnel = 28.3 ± 0.7 g, Day 21: ***χ***^2^ = 3.66, *P* = 0.06). Combining all handling and behavioural tests, mice that were tail handled defecated more often than mice that were tunnel handled (***χ***^2^ = 8.33, *P* = 0.004).

## Discussion

Our study demonstrates that, in the most part, the benefits of tunnel handling to reduce anxiety in laboratory mice persist after repeated restraint and procedures. Tail handled mice showed greater reluctance to interact with a handler, and greater levels of anxiety than tunnel handled mice in standard behavioural tests, and these effects persisted after experience of repeated restraint, IP injections or anaesthesia. Our results also suggest that repeated restraint was more aversive than being picked-up by the respective handling methods, as mice that underwent repeated restraint interacted less with their handler, compared with mice that had experienced handling only. However, after repeated restraint, tunnel handled mice continued to show significantly greater interaction with a handler and were more explorative in behavioural tests compared with tail handled mice. The recency and type of procedure also affected how the different handling methods influenced the behaviour of mice. The day following IP injections and anaesthesia, tunnel handled mice spent significantly more time interacting with the handler than tail handled mice, compared with when interaction with the handler was measured immediately after procedures. In addition, tunnel handled mice that underwent anaesthesia spent significantly more time interacting with the handler than those that underwent IP injection. These results suggest that both restraint and procedures influence the behavioural responses of mice. Despite this, tunnel handling significantly increased interaction with a handler within 24 h of both procedures, and in general, increased exploration in standard behavioural tests, compared with tail handed mice. Overall, this suggests that tunnel handling can improve the welfare of mice and reduce handling related stress, even when experimental protocols require the use of putatively more aversive procedures.

As part of experimental protocols mice often undergo multiple procedures that can cause pain or discomfort and require handling and restraint; including injection and anaesthesia. Our study is the first to investigate how repeated IP injections influence the benefits of non-aversive handling. This study adds to evidence from multiple mouse strains that a single scruff restraint, and lifting the tail for abdominal inspection, where the mouse is held in place by the tail but not lifted by the tail, does not negate the benefits of tunnel handling upon voluntary interaction with a handler^9^. Similarly, Nakamura and Suzuki^12^ have shown that tunnel handling increased voluntary interaction with a handler and ease of handling (rating scale for wildness^25^) after repeated daily restraint and oral gavage of saline. This study also showed that tunnel handled mice showed greater exploration in an open field test and elevated plus maze following a single IP injection in comparison with tail handled mice^12^. Most recently, Hurst and Gouveia^16^ have shown that repeated scruff restraint and subcutaneous injection did not reduce the positive impact of tunnel handling upon reducing handling stress^16^. In this study, we have substantially advanced previous findings, and replicated results at another institution. Taken together these results strongly suggest that picking the mouse up by the tail increases behavioural anxiety and aversion to a handler, even when mice undergo repeated restraint or other procedures.

Laboratory mice can be required to undergo short periods of anaesthesia to implant devices for substance delivery^18^, or for physiological monitoring and recording^26,27^. Anaesthesia commonly has to be repeated to image the development of diseases^28^, or inflammation due to surgery or other potentially harmful procedures^17,19^. Isoflurane has specifically been shown to be aversive to mice^29^ and can negatively impact mouse welfare^20^, especially when administered repeatedly. Here we have shown that the positive impacts of tunnel handling upon voluntary interaction with a handler and reduced anxiety in standard behavioural tests persists after repeated short duration (5 min) anaesthesia with isoflurane. This is a novel finding that suggests the methods used to pick up mice, rather than experience of anaesthesia, causes aversion to a handler and increases anxiety.

In agreement with previous studies we found that the impact of handling methods upon behaviour was evident within two weeks of daily handling^9,10,12,14^. Tail handled mice interacted less with the handler after only five days of daily handling and showed greater levels of anxiety compared to tunnel handled mice after nine days of daily handling. We have also shown that tail handled mice continued to interact less with the handler after 4–8 weeks of once a week handling during cage cleaning between the experiments. Furthermore, the effects of handling upon voluntary interaction persisted for the duration of the experiments, which were 3–5 months in duration. This suggests that tunnel handling does not need be conducted daily to have a positive impact upon the behaviour of mice and that these beneficial effects are persistent. The duration of handling by tail or tunnel methods (2–60 s) has recently been shown to not influence the beneficial effects of tunnel handling, and tunnel handling for as little as four fortnightly cage cleans, has also been shown to substantially increase voluntary interaction with a handler compared with tail handled mice^16^. Our findings confirm the rapid behavioural effects of handling upon mice and show that brief experience of tunnel handling during cage cleaning is sufficient to maintain the beneficial impact of tunnel handling upon indicators of handling stress.

Unlike previous studies, handling method did not influence voluntary interaction with a handler on the first day of handling in Experiment 1. Most previous studies have measured the time spent by mice interacting with the handler holding the handling device; i.e. a gloved hand for tail and cup methods, or a hand holding the home cage tunnel for the tunnel method^9,12,14^ (but see Roughan and Sevenoaks^17^). Our methods differed from previous studies as we used interaction with the handler’s hand for both tail and tunnel handled mice. While investigating how mice interact with the instrument that is being used to handle them is insightful and relevant in practise, mice show positive thigmotaxis, preferring to hide in narrow crevices when disturbed^9^. Therefore, mice are more likely to interact with a tunnel than with a hand regardless of handling techniques. This may explain why, in previous studies, after a single handling session mice showed a significant preference for the tunnel compared with the handler’s hand^9,14^. To investigate this potential confound we used the same stimulus for both tunnel and tail handled mice and confirmed that tail handled mice interacted less with the handler compared to tunnel handled mice, consistent with previous studies. However, in contrast to previous findings^16^, we found that repeated restraint caused mice to interact less overall with a handler than mice that had only been picked up by their respective handling method. We also did not find evidence that the Head Support Restraint method was less aversive than the more commonly used Pinch Restraint. Notably, restraint is necessary for health checks and for conducting procedures, so is essential for experiments and ensuring the health of mice, while tail handling is not. Therefore, tunnel handling is a refinement that can improve the welfare of mice that undergo repeated restraint and procedures.

Whether voluntary interaction was measured immediately after the procedure or the day after, and the procedure type (IP injection vs. anaesthesia) influenced how the handling method affected willingness to interact with a handler. Tunnel handled mice spent significantly more time interacting with the handler than tail handled mice on the day following the procedure, compared with immediately after the procedure. Also, tunnel handled mice that underwent anaesthesia spent significantly more time interacting with the handler than those that underwent IP injection. In contrast, Hurst and Gouveia^16^ did not find that subcutaneous injection or restraint diminished voluntary interaction with a handler. The different methods used to assess voluntary interaction may have contributed to these contrasting results. We also found that handling method did not influence time spent on the open arm of the EPM for mice that underwent IP injections. This further suggests that the type of procedure experienced can influence how handling methods affect the behaviour of mice. Compared with previous studies mice visited the open arm of the EPM less often and spent a greater amount of time on the open arm in all experiments. In addition, there was no significant difference in time spent in the centre of the OFT between handling methods. Many variables can influence behavioural responses in the EPM and OFT, including housing conditions and illumination levels between laboratories^30,31^, which may have contributed to the difference between our results and previous studies. Importantly, as the impacts of handling method persisted after repeated scruff restraint, were evident within 24 h after both IP injection and anaesthesia, and caused a difference in behaviour in standard behavioural tests our results ultimately correspond with previous studies^9,12,16^.

Our results suggest that repeated restraint is aversive for laboratory mice, and that the type of procedure experienced, and duration after which behaviour is measured post-procedure affects the willingness of mice to interact with a handler. Yet, tunnel handling reduced anxiety in standard behavioural tests and increased willingness to interact with a handler, compared with tail handling, even after repeated restraint and procedures. Therefore, the simple yet effective refinement of using a tunnel rather than the tail to pick up mice, can significantly improve animal welfare and has the potential to improve scientific data quality. Accumulating evidence shows that tunnel handling reduces anxiety and increases willingness to interact with a handler even after brief handling and continues to have a positive impact after repeated restraint and procedures, that are commonly used in the laboratory. In conclusion, evidence suggests mice should be handled using a tunnel and not by their tails.

## Supplementary information

Supplementary Information 1.

Supplementary Information 2.

Supplementary Information 3.
