# Supplementary material for: Benefits of tunnel handling persist after repeated restraint, injection and anaesthesia
Source: Sci Rep. 2020 Sep 3;10:14562. doi: 10.1038/s41598-020-71476-y (PMC7471957; doi:10.1038/s41598-020-71476-y)
Supplement: Supplementary file 2 — Supplementary Information 2. [file 41598_2020_71476_MOESM2_ESM.docx]

**Benefits of tunnel handling persist after repeated restraint, injection and anaesthesia**

Lindsay J. Henderson*^a,b,c^, Bridgette L. Dani^b^, Michelle N. Serrano^b^, Tom V. Smulders^a,b^ & Johnny V. Roughan^b^

^a^Centre for Behaviour and Evolution, ^b^Institute of Neuroscience, Newcastle University, Newcastle upon Tyne, NE2 4HH, UK

^c^The Roslin Institute, The University of Edinburgh, Midlothian EH25 9RG

*Corresponding author: Lindsay.Henderson@roslin.ed.ac.uk

**Supplementary Materials 1.**

**Table 1 Overview of A) Experiment 1 and B) Experiments 2 and 3.**

| **A) Experiment 1** |  |  |  |  |  |
| --- | --- | --- | --- | --- | --- |
| **Data** | **Dependent Variable** | **Statistical test/ Error structure/ Transformation** | **Factors**  **fixed/ random** | **Experimental unit** | **Sample size** |
| Voluntary interaction test – all days (1, 5 and 9) | ﻿Percentage time spent interacting | GLMM/  Gaussian/  Square root | Handling method (2 levels; tail, tunnel); Day (3 levels; Day 1, 5 and 9); Order (2 levels; pre or post handling)  / Cage | Cage | N = 12 tail handled  N = 12 tunnel handled |
| Voluntary interaction test – day 9 | ﻿Percentage time spent interacting | GLM/  Gaussian/  Square root | Handling method (2 levels; tail, tunnel); Treatment (3 levels; handling only, pinch restraint, head support restraint) | Cage | N = 12 tail handled  N = 12 tunnel handled |
| Elevated plus maze | ﻿Number of open arm entries; Duration on open arms (%) | GLMM/  Poisson/  None;  GLMM/  Gaussian/  Arcsine | Handling method (2 levels; tail, tunnel); Treatment (3 levels; handling only, pinch restraint, head support restraint)  / Cage | Mouse | N = 24 tail handled  N = 24 tunnel handled |
| Open field test | ﻿Number of entries to centre;  Duration in centre (%) | GLMM/  Poisson/  None;  GLMM/  Gaussian/  Square root | Handling method (2 levels; tail, tunnel); Treatment (3 levels; handling only, pinch restraint, head support restraint)  / Cage | Mouse | N = 24 tail handled  N = 24 tunnel handled |
| Defecation | Total number of defecations | GLMM/  Poisson/  None | Handling method (2 levels; tail, tunnel)  / Cage | Mouse | N = 24 tail handled  N = 24 tunnel handled |
| Mass day 0 or day 10 | Mass (g) | GLM/  Gaussian/  None | Handling method (2 levels; tail, tunnel) | Mouse | N = 24 tail handled  N = 24 tunnel handled |

| **B) Experiments 2 & 3** |  |  |  |  |  |
| --- | --- | --- | --- | --- | --- |
| **Data** | **Dependent Variable** | **Statistical test/ Error structure/ Transformation** | **Factors**  **Fixed**  **/ random** | **Experimental unit** | **Sample size** |
| Handling only – Voluntary interaction tests day 1 and 5 | Percentage time spent interacting | GLMM/  Gaussian/  Square root | Handling method (2 levels; tail, tunnel); Day (2 levels; 1 and 5)  / Cage | Cage | N = 11 tail handled  N = 11 tunnel handled |
| Handling only –  open field test | Number of entries to centre; Duration in centre (%) | GLMM/  Poisson/  None;  GLMM/  Gaussian/  Arcsine | Handling method (2 levels; tail, tunnel)  / Cage | Mouse | N = 22 tail handled  N = 22 tunnel handled |
| Procedures – Voluntary interaction tests | Percentage time spent interacting | GLMM/  Gaussian/  Arcsine | Handling method (2 levels; tail, tunnel); Procedure (2 levels; IP injection or anaesthesia); Day (2 levels; day of procedure or day after procedure)  / Cage | Cage (mean from three procedures) | *IP injection*  N = 5 tail handled  N = 5 tunnel handled  *Anaesthesia*  N = 6 tail handled  N = 6 tunnel handled |
| Procedures – elevated plus maze | Number of open arm entries; Duration on open arms (%) | GLMM/  Poisson/  None;  GLMM/  Gaussian/  Square root | Handling method (2 levels; tail, tunnel); Procedure (2 levels; IP injection or anaesthesia)  / Cage | Mouse | *IP injection*  N = 10 tail handled  N = 10 tunnel handled  *Anaesthesia*  N = 12 tail handled  N = 12 tunnel handled |
| Defecation | Total number of defecations | GLMM/  Poisson/  None | Handling method (2 levels; tail, tunnel)  / Cage | Mouse | N = 20 tail handled  N = 24 tunnel handled |
| Mass day 0 or day 21 | Mass (g) | GLM/  Gaussian/  None | Handling method (2 levels; tail, tunnel) | Mouse | N = 22 tail handled  N = 22 tunnel handled |
