# Supplementary material for: Benefits of tunnel handling persist after repeated restraint, injection and anaesthesia
Source: Sci Rep. 2020 Sep 3;10:14562. doi: 10.1038/s41598-020-71476-y (PMC7471957; doi:10.1038/s41598-020-71476-y)
Supplement: Supplementary file 3 — Supplementary Information 3. [file 41598_2020_71476_MOESM3_ESM.docx]

**Benefits of tunnel handling persist after repeated restraint, injection and anaesthesia**

Lindsay J. Henderson*^a,b,c^, Bridgette L. Dani^b^, Michelle N. Serrano^b^, Tom V. Smulders^a,b^ & Johnny V. Roughan^b^

^a^Centre for Behaviour and Evolution, ^b^Institute of Neuroscience, Newcastle University, Newcastle upon Tyne, NE2 4HH, UK

^c^The Roslin Institute, The University of Edinburgh, Midlothian EH25 9RG

*Corresponding author: Lindsay.Henderson@roslin.ed.ac.uk

**Table 2.** Experiment 1. GLMMs investigating the effect of handling method (tail or tunnel) and restraint treatment (handling only, Pinch Restraint, Head Support Restraint) upon behaviour in the Elevated Plus Maze (EPM) and the Open Field Test (OFT). Cage was included as a random factor to avoid pseudoreplication. N = 48.

| **Factor** |  |  |  |
| --- | --- | --- | --- |
| **EPM** | ***χ^2^*** | ***d.f.*** | ***P*** |
| ***Entries onto open arm*** |  |  |  |
| Handling | 20.99 | 1 | **< 0.001** |
| Treatment | 2.31 | 2 | 0.32 |
| Handling x Treatment | 1.48 | 2 | 0.48 |
| ***Proportion time on open arm*** |  |  |  |
| Handling | 4.28 | 1 | **0.04** |
| Treatment | 3.12 | 2 | 0.21 |
| Handling x Treatment | 0.01 | 2 | 0.99 |
| **OFT** | ***χ^2^*** | ***d.f.*** | ***P*** |
| ***Entries into centre*** |  |  |  |
| Handling | 12.62 | 1 | **< 0.001** |
| Treatment | 0.66 | 2 | 0.72 |
| Handling x Treatment | 0.25 | 2 | 0.88 |
| ***Proportion time in centre*** |  |  |  |
| Handling | 1.99 | 1 | 0.16 |
| Treatment | 0.01 | 2 | 0.99 |
| Handling x Treatment | 0.96 | 2 | 0.62 |

**Table 3.** Experiment 1. GLM investigating the effect of handling method (tail or tunnel) upon mass (g) at the beginning of the experiment (day 0) and after the experiment was completed (day 10). B) GLMM investigating the effect of handling method (tail or tunnel) upon number of total defecations produces during handling and behavioural observations. N = 48.

| **A) Mass** | ***t*** | ***d.f.*** | ***P*** |
| --- | --- | --- | --- |
| ***Day 0*** |  |  |  |
| Sex | 7.70 | 1 | **< 0.001** |
| Handling | 1.08 | 1 | 0.29 |
| ***Day 10*** |  |  |  |
| Sex | 8.02 | 1 | **< 0.001** |
| Handling | 1.30 | 1 | 0.20 |
| **B) Defecation** | ***χ^2^*** | ***d.f.*** | ***P*** |
| Handling | 8.70 | 1 | **0.003** |

**Table 4.** Experiment 2 & 3. GLMM investigating the effect of handling method (tail or tunnel) and day (1 or 5) upon voluntary interaction with a handler (percentage of time). N = 22. B) GLMM investigating the effect of handling method (tail or tunnel) upon behaviour in the Open Field Test (OFT). Cage was included as a random factor to avoid pseudoreplication. N = 44.

| **Factor** |  |  |  |
| --- | --- | --- | --- |
| **A) Voluntary interaction** | ***χ^2^*** | ***d.f.*** | ***P*** |
| Handling | 17.14 | 1 | **< 0.001** |
| Day | 27.67 | 1 | **< 0.001** |
| Handling x Day | 1.12 | 1 | 0.29 |
| **B) OFT** | ***χ^2^*** | ***d.f.*** | ***P*** |
| ***Entries into centre*** |  |  |  |
| Handling | 5.49 | 1 | **0.02** |
| ***Proportion time in centre*** |  |  |  |
| Handling | 3.26 | 1 | 0.07 |

**Table 5.** Experiment 2 & 3. A) GLMM investigating the effect of handling method (tail or tunnel) and day (day of or day after procedure), procedure (anaesthesia or IP injection) and their interactions upon voluntary interaction with a handler (percentage of time). N = 22. B) GLMM investigating the effect of handling method (tail or tunnel) and procedure (anaesthesia or IP injection) upon behaviour in the Elevated Plus Maze (EPM). Cage was included as a random factor to avoid pseudoreplication. N = 44.

| **Factor** |  |  |  |
| --- | --- | --- | --- |
| **A) Voluntary interaction** | ***χ^2^*** | ***d.f.*** | ***P*** |
| Handling | 16.15 | 1 | **< 0.001** |
| Day | 35.49 | 1 | **< 0.001** |
| Procedure | 17.86 | 1 | **< 0.001** |
| Handling x Day | 8.92 | 1 | **< 0.01** |
| Handling x Procedure | 3.97 | 1 | 0.05 |
| Day x Procedure | 0.06 | 1 | 0.81 |
| Handling x Day x Procedure | 0.03 | 1 | 0.85 |
| **B) EPM** | ***χ^2^*** | ***d.f.*** | ***P*** |
| ***Entries onto open arm*** |  |  |  |
| Handling | 8.37 | 1 | **0.004** |
| Procedure | 1.14 | 1 | 0.29 |
| Handling x Procedure | 0.58 | 1 | 0.45 |
| ***Proportion time on open arm*** |  |  |  |
| Handling | 9.44 | 1 | **0.002** |
| Procedure | 2.75 | 1 | 0.10 |
| Handling x Procedure | 3.80 | 1 | 0.05 |

**Table 6.** Experiment 2 &3. A) GLM investigating the effect of handling method (tail or tunnel) upon mass (g) at the beginning of the experiment (day 0) and after the experiment was completed (day 21). B) GLMM investigating the effect of handling method (tail or tunnel) upon number of total defecations produces during handling, procedures and behavioural observations. N = 44.

| **A) Mass** |  |  |  |
| --- | --- | --- | --- |
| ***Day 0*** | ***t*** | ***d.f.*** | ***P*** |
| Sex | 34.93 | 1 | **< 0.001** |
| Handling | 0.97 | 1 | 0.32 |
| ***Day 21*** |  |  |  |
| Sex | 26.60 | 1 | **< 0.001** |
| Handling | 3.65 | 1 | 0.06 |
| **B) Defecation** | ***χ^2^*** | ***d.f.*** | ***P*** |
| Handling | 8.33 | 1 | **0.004** |
